# Histological evaluation of three-dimensional rectangular grid plates in treatment of mandibular fractures: an experimental study

**DOI:** 10.1186/s12903-026-09946-4

**Published:** 2026-10-17

**Authors:** Mohamed Mohsen Elbanna, Ibrahim H. Ahmed, Mohamed Hassan Eid, Mohamed Elsholkamy, Mohamed Said Hamed

**Affiliations:** 1 Faculty of Dentistry, Suez Canal University, Ismailia, Ismailia Egypt; 2 Faculty of Veterinary Medicine, Suez Canal University, Ismailia, Egypt; 3 Faculty of Dentistry, Suez Canal University, Ismailia, Egypt

**Keywords:** Mandibular fractures, 3D rectangular grid plates, Locking plates, Histomorphometric analysis

## Abstract

**Aim of the work:**

Evaluate the efficacy of 3D rectangular grid plates in treatment of mandibular fractures in comparison to the more commonly used locking plates.

**Subjects and methods:**

This experimental study was conducted on sixteen mature male Mongrel dogs (ranged from 1-2 years old), weighting about 15-20 kg each. A linear fracture was done using surgical sagittal saw with aid of mallet and chisel in the right side of the mandible in the premolar molar area for each animal of the study under general anesthesia. The animals were divided into two groups: Group A was fixated with 3D rectangular grid plate and six 7 mm monocortical screws and Group B was fixated with two locking plates; the inferior plates fixated with 11 mm bicortical screws and the superior one fixated with 7 mm monocortical screws. The bone formation and remodeling is assessed using histomorphometric analysis of bone biopsy.

**Results:**

Group A subjects showed significantly less bone formation than group B (26.78±4.3 vs 48.4±10.3 respectively in postoperative week 4 *P*<0.005 and 28.119±10.507 vs 51.109±11.348 respectively in postoperative week 12 *P*<0.005). The surface area of the fracture was also significantly lower in group A than in group B (0.317 ± 0.065 vs 0.485 ± 0.105 respectively in postoperative week 4 *P*<0.005 and 0.287 ± 0.105 vs 0.511 ± 0.115 respectively in postoperative week 12 *P*<0.005).

**Conclusions:**

Fixation with 3D rectangular grid plates demonstrated a bone healing pattern characterized by closer fracture segment approximation and reduced callus formation, consistent with primary bone healing, as evidenced by histological and histomorphometric analysis, compared with locking plates.

## Introduction

Despite being the largest and strongest face bone, the mandible is the most common facial fracture after the nasal bone because to its prominence, anatomic structure, mobility, and lack of bone support. Mandibular fractures can induce malocclusion, joint dysfunction, discomfort, infection, and paresthesia, as well as destabilize the airway [1].

Historically, closed reduction using maxillomandibular fixation (MMF) was the primary treatment modality, but limitations such as prolonged immobilization and patient discomfort have led to widespread adoption of open reduction and internal fixation (ORIF). ORIF techniques allow direct fracture visualization and stable fixation, facilitating early mandibular function and improved patient outcomes. Among these, miniplate fixation based on the principles of load-sharing along ideal lines of osteosynthesis has become a widely accepted standard for many mandibular fracture patterns, particularly in the body and angle regions [2].

Contemporary fixation systems include single conventional miniplates, double miniplates, locking plates, and three-dimensional (3D) or rectangular grid plates, each designed to enhance stability against functional forces and improve bone healing. Single miniplates offer simplicity and reduced hardware, whereas double plates and locking designs provide increased rigidity in comminuted or high-load fractures. Three-dimensional grid plates, in particular, aim to resist torsional and bending forces through a triangulated configuration, potentially improving clinical outcomes in challenging fracture sites such as the mandibular angle [3].

Locking plate systems have been increasingly used in mandibular fracture fixation because they provide enhanced stability by creating a fixed-angle construct between the plate and screws, thereby reducing dependence on plate–bone contact and minimizing screw loosening, particularly in cases of poor bone quality, comminution, or atrophic mandibles. However, the increased rigidity associated with locking plates may result in stress shielding and reduced interfragmentary strain, which can adversely affect physiological bone healing and lead to delayed remodeling or excessive hardware-related complications. In addition, locking plate systems are associated with increased cost, greater hardware bulk, and longer operative time, especially when more than one plate is used [4].

A common biomechanical misconception regarding rectangular 3D grid plates is that their geometric complexity or perceived rigidity may compromise physiological fracture healing or inadequately control functional mandibular forces. In fact, the three-dimensional box-like configuration of rectangular grid plates provides simultaneous resistance to bending, torsional, and shear forces, which are dominant during mandibular function. Unlike conventional single linear miniplates that primarily counter forces along one axis, the interconnected horizontal and vertical struts of the rectangular grid enable multidirectional load distribution, reducing stress concentration at the fracture line and individual screw–bone interfaces. This biomechanical behavior allows appropriate fracture segment support while maintaining favorable interfragmentary strain, thereby promoting primary bone healing without excessive callus formation. Consequently, concerns regarding insufficient biomechanical performance largely arise from limited familiarity with 3D load-sharing mechanics rather than inherent mechanical shortcomings of the rectangular grid design [5]. Most previous studies have primarily focused on the biomechanical stability and mechanical performance of these fixation methods. However, limited information is available regarding their influence on the biological aspects of bone healing, particularly at the histological and histomorphometric levels. The comparative effects of conventional locking plates and three-dimensional rectangular grid plates on new bone formation, callus development, and bone microstructure remain insufficiently investigated.

The present study aims to compare the effects of conventional locking plates and three-dimensional rectangular grid plates on mandibular fracture healing, using histological and histomorphometric analysis to evaluate new bone formation, callus development, and microstructural bone quality, in order to determine differences in the biological healing response associated with each fixation method.

## Subjects and methods

The study protocol was revised and approved by the Institutional Animal Care and Use Committee of Suez Canal University. All ethical considerations involving experimental animals were followed by the researcher, and a pledge form for animal care was submitted to the Research Ethics Committee (REC) before commencing the study. The committee operates in accordance with the principles of the ARRIVE Guidelines 2.0 to ensure appropriate reporting and ethical conduct of animal research.

The study was conducted on sixteen mature male Mongrel dogs (ranged from 1 to 2 years old), weighting about 15–20 kg each. The dogs were selected from animal house in Faculty of Veterinary Medicine, Suez Canal University. The use of a homogenous animal population was intended to minimize biological variability and ensure consistent baseline bone quality. This allowed clearer interpretation of differences attributable to the fixation systems themselves. They were clinically examined to rule out the presence of any disease and kept under clinical observation for three weeks preoperatively and fed cooked meat, bread, milk, and water. The animals were housed in separate cages, supplied with food and a bucket for water and allowed to live in optimal conditions.

The number of animals used in this study was determined in accordance with the ethical guidelines and restrictions set by the Institutional Animal Care and Use Committee, which limits the use of large animals to the minimum required achieving scientific validity. The final sample size of 8 animals per group is consistent with previously published experimental canine mandibular fracture models [6, 7].

**Fig. 1:**
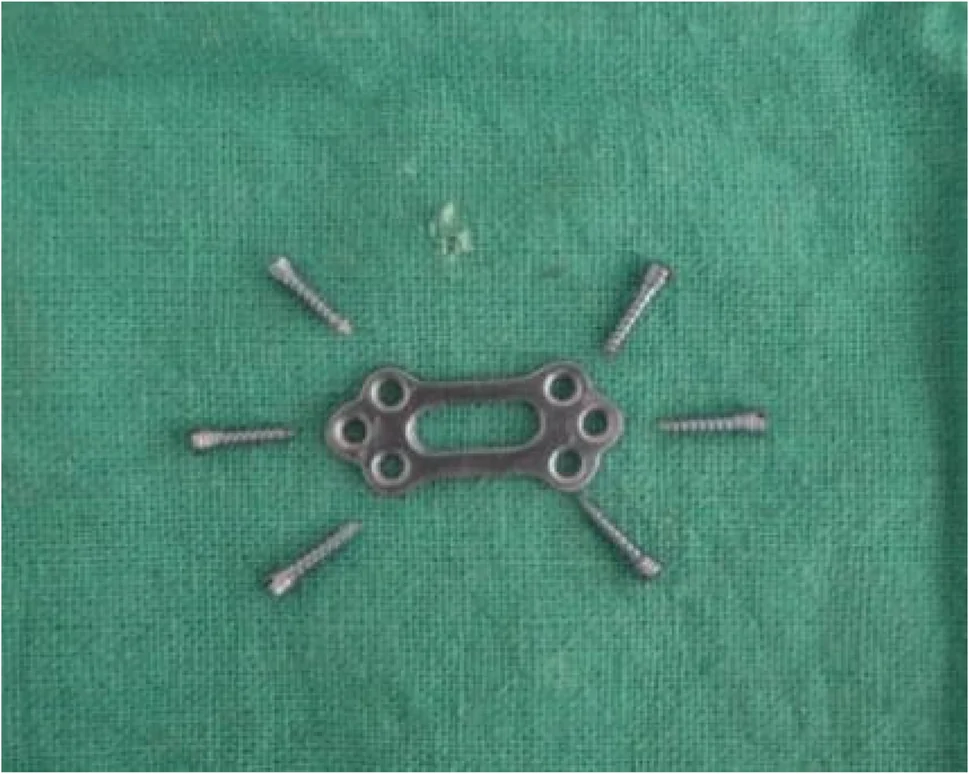
3D Rectangular Grid Plate (a) with 7 mm monocortical screws

**Fig. 2:**
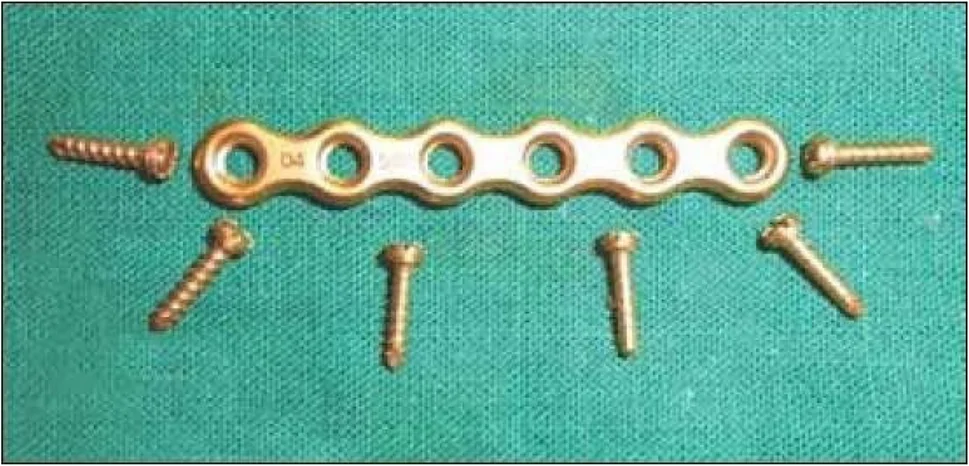
Locking plates (b) with 11 mm bicortical and 7 mm monocortical screws

A linear fracture was done using surgical sagittal saw with aid of mallet and chisel in the right side of the mandible in the premolar molar area for each animal of the study under general anesthesia. The animals were divided into two groups:

- Group A: included 8 dogs were fixated with 3D rectangular grid plate and six 7 mm monocortical screws (Fig. 1)
- Group B: included 8 dogs were fixated with two locking plates; the inferior plates fixated with 11 mm bicortical screws and the superior one fixated with 7 mm monocortical screws (Fig. 2)

### Preoperative preparation

This was done by spraying the cages with 6/1000 ml diazinon (c) and the dogs were bathed with 1/1000 diazinon. They were injected by Ivermectin (d) 1 ml/50kg body weight subcutaneously to guard against ectoparasytic and endoparasytic infestations. 6–8 h before the operation food and water were kept away from the dogs to prepare them for anesthesia. Animals were weighed to calculate the dosage of the drugs used. Each animal was premedicated by administration of Midazolam (Midathetic) in a dose of 0.1 mg/kg body weight 10–20 min prior to surgery. Animals were cannulated using 20 gauge IV cannula. Induction and maintainance of anesthesia was done by IV administration of sodium thiopental 2.5% solution 20–30 mg/kg via intravenous cannula, one third of the administration dose was injected within 15 s and the remainder was given slowly till loss of the pedal and corneal reflexes, constriction of the pupils, and development of shallow regular respiration.

### The surgical procedure

**Fig. 3:**
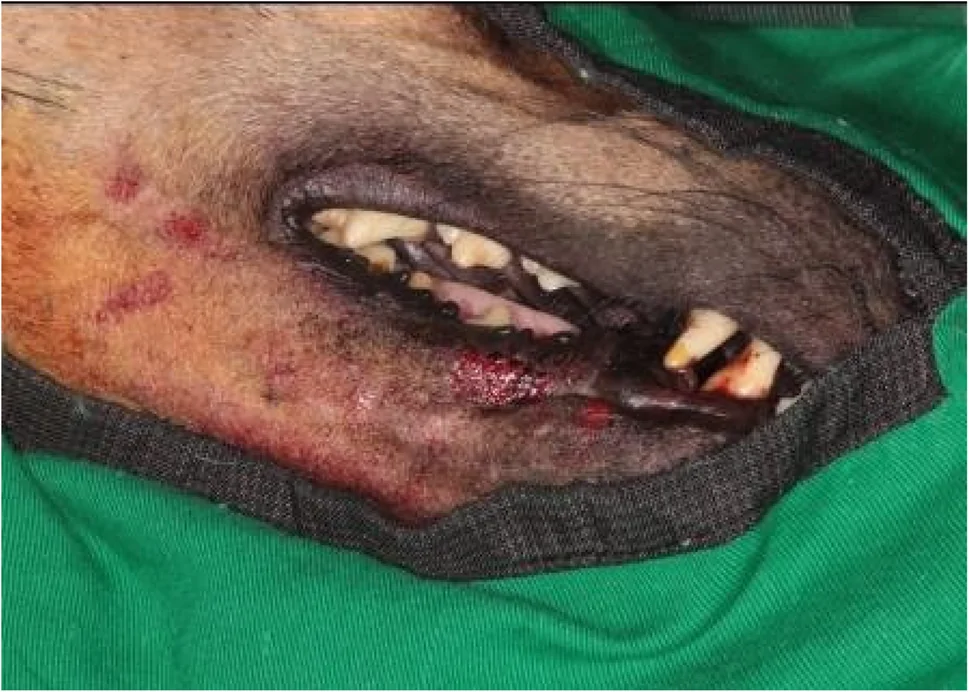
Preoperative field of prepared dogs

The surgery was performed under aseptic conditions and the surgical field was scrubbed intraorally and circumorally by using Betadine surgical scrub. (Fig. 3) the procedure was done by a single experienced team to ensure uniform technique and minimize procedural variability that may affect the results of the study.

**Fig. 4:**
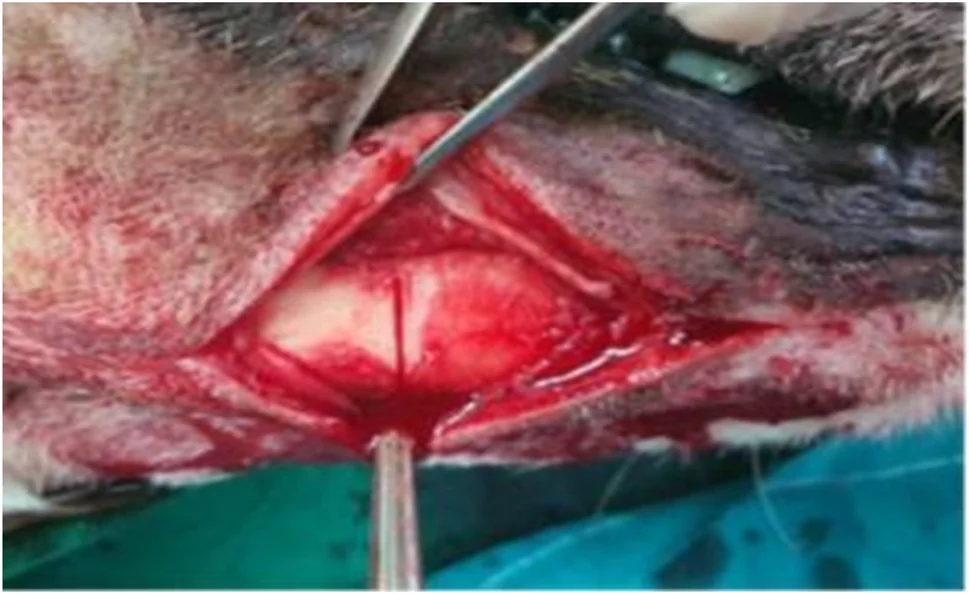
Surgical field showing fracture line done by surgical saw

**Fig. 5:**
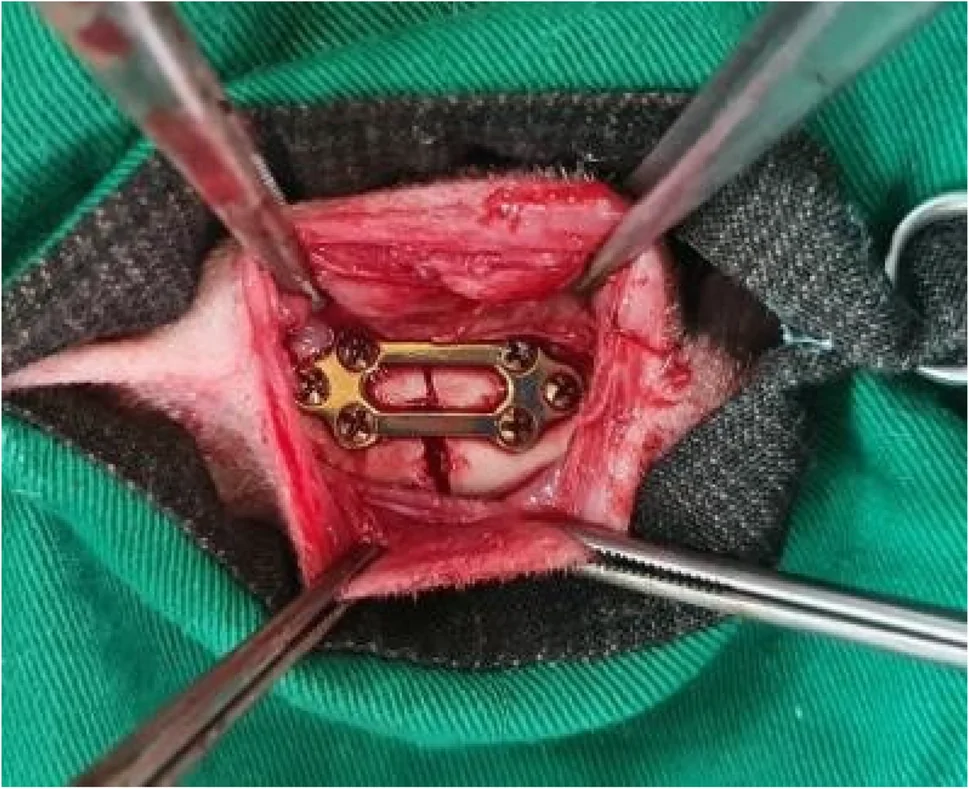
Intraoperative fixation with 3D rectangular grid plates

**Fig. 6:**
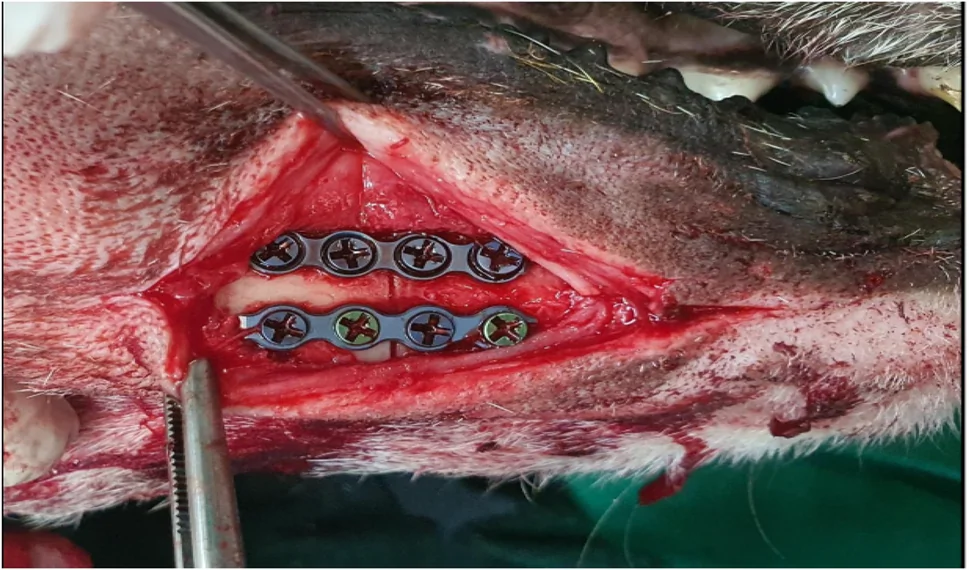
Fixation of mandibular fracture using 2 locking plates

**Fig. 7:**
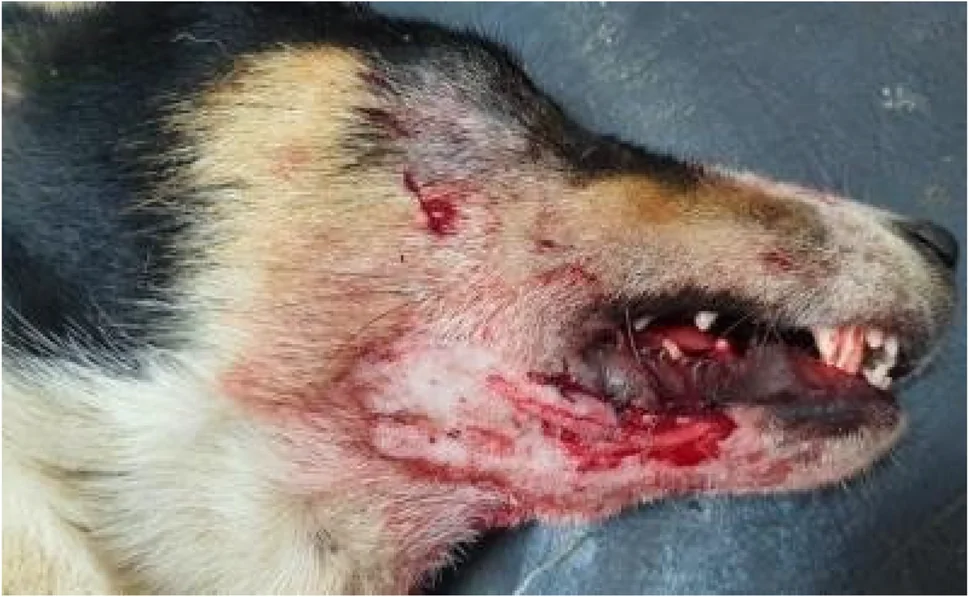
Surgical field after suturing

1. Local anesthesia with vasoconstrictor was administered in the surgical field for hemostasis and to reduce postoperative pain.
2. In the right side of the mandible extraoral incision using blade number 10 was made in the premolar molar area.
3. The flap was reflected using periosteal elevator until clear exposure of the surgical site occurred.
4. Surgical sagittal saw with irrigation was used to make a linear fracture cut in the premolar molar area with aid of mallet and chisel to separate the fractured segments. (Fig. 4) The premolar–molar region was chosen because it is a biomechanically relevant and frequently fractured site, making it appropriate for evaluating fixation device performance.
5. The fracture site was fixated with 3D rectangular grid plate with 7 mm monocortical screws or in group A or fixated with dual-locking plates with 11 mm bicortical and 7 mm monocortical screws in group B. The plate is aligned parallel to the occlusal plane and positioned 3–4 mm dorsal to the ventral mandibular border, which is consistent with published biomechanical guidelines for canine mandibular fracture stabilization [5]. Screw trajectories are directed away from the mandibular canal and tooth roots, maintaining the recommended safe corridors for monocortical and bicortical engagement. The construct spans the entire fracture line with symmetrical screw distribution on each side, ensuring adequate load transfer across the defect and minimizing risk of shear or torsional instability. This placement adheres to established principles that emphasize stabilization along the tension surface to counteract bending forces generated during mastication and to maintain optimal mechanical environment for bone healing [8] (Figs. 5 and 6).
6. Finally, in both groups, the surgical wound was primarily closed with interrupted sutures (0 Vicryl) (Fig. 7)

### Postoperative care and follow up

After the operation the dogs were allowed to recover in a warm place with dry non slippery floor. The dogs were given IM Cefotaxime 500 mg twice daily for seven successive days to prevent postoperative infection along with IM Diclofinac sodium 75 mg twice daily for three successive days to control pain and inflammation. Postoperative procedures were carefully standardized following established veterinary protocols to ensure consistency across subjects. All animals were monitored daily, and adherence to the standardized dietary regimen was strictly maintained. The dogs were examined daily for any late wound complications or delayed reactions and their appetite, weight, and general condition and kept on soft diet for one week then fed normal diet until the end of the study.

### Animal euthanasia

The dogs were euthanized at 4 and 12 weeks after surgery (4 per group). The dogs were painlessly euthanized by administration of an overdose of thiopental through cannula. These time points were chosen because they represent the critical phases of early callus formation and intermediate healing in bone in order to provide strong insight into the primary healing behavior of the fixation systems.

### Histological preparation of the sample

Soon after sacrifice, the mandibular specimens were placed in 10% neutral buffered formalin for 24 h for primary fixation. Histological sections were obtained from the fracture line located in the premolar–molar region of the mandible, corresponding to the site of plate fixation. Samples were taken from the central portion of the fracture gap to ensure consistency and avoid adjacent intact bone or soft tissue. The specimens were then decalcified, dehydrated with ethanol, cleared with xylene, and embedded in paraffin wax. The fracture site was sectioned serially using a microtome at a thickness of 5 μm, followed by staining with hematoxylin and eosin (H&E) according to the procedure outlined by Suvarna et al [9]. Sections were examined under light microscopy to assess new bone formation, callus development, and tissue organization at the fracture site. The assessments were performed by a single experienced histopathologist using standardized criteria to minimize subjective bias.

The quantitative histomorphometric evaluation was conducted using image analysis. The data was collected through the Leica Qwin 500 image analyzer system (England), which included a color video camera, monitor, and an IBM personal computer linked to the Olympus BX 40 microscope (Olympus, Japan), all operated by Leica Qwin 500 software. The system was automatically calibrated to convert the image analyzer’s pixel measurements into micrometer units. The area of newly formed bone was measured in ten fields, and the data was recorded in tables for statistical analysis. The results were presented as means and standard deviation (SD), and statistical significance was assessed using SPSS version 22 software (IBM SPSS). A p-value of ≤ 0.05 was considered statistically significant. To assess the adequacy of the sample size used in this study (*n* = 8 per group), a post hoc power analysis was performed for all primary outcomes using G*Power software (version 3.1.9.7). The analysis was based on an alpha level of 0.05and the observed effect sizes (Cohen’s 𝑑).

## Results

**Fig. 8:**
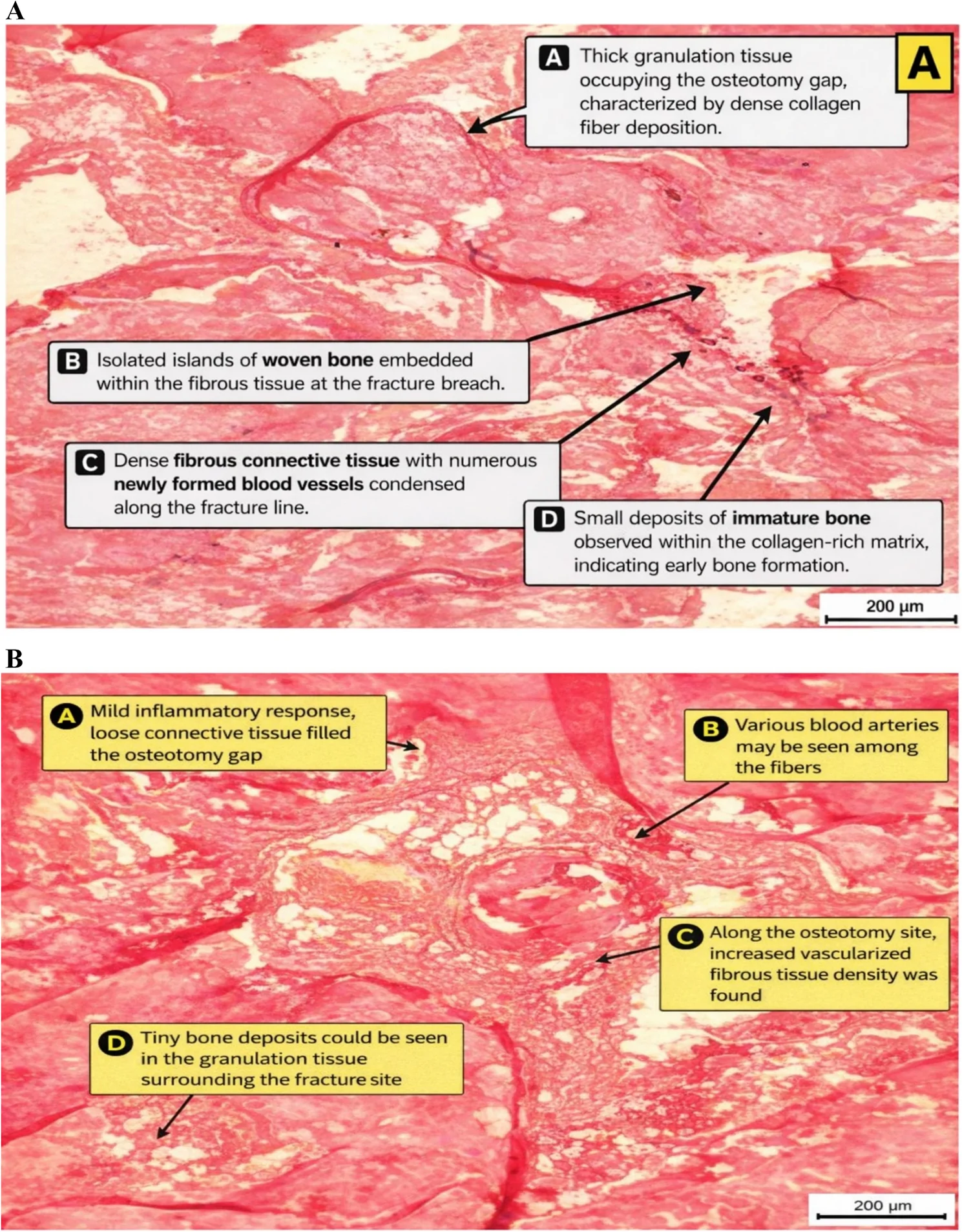
**A** Histological evaluation of bone healing at the fracture site at 4 weeks in group A. Histological sections stained with Hematoxylin and Eosin (H&E). Images were captured at ×100 magnification. Orientation: cranial aspect at the top, caudal aspect at the bottom. **B** Histological evaluation of bone healing at the fracture site at 4 weeks in group B. Histological sections stained with Hematoxylin and Eosin (H&E). Images were captured at ×100 magnification. Orientation: cranial aspect at the top, caudal aspect at the bottom

The dog’s specimens were subjected to Histopathological examination. These specimens were taken at different interval 4 and 12 weeks postoperative. The results of our study after 4 weeks postoperative show that in group A subjects there was thick granulation tissue with more collagen fibers than usual in the osteotomy gap. The osteotomy’s breach revealed the implantation of isolated islands of woven bone within the fibrous tissue. A dense fibrous tissue with blood vessels condensed along the fracture line, and tiny immature bone deposits could be seen inside the collagen fibers. (Fig. 8A) On the other hand group B showed a mild inflammatory response, loose connective tissue filled the osteotomy gap. Various blood arteries may be seen among the fibers. Along the osteotomy site, increased vascularized fibrous tissue density was found. Tiny bone deposits could be seen in the granulation tissue surrounding the fracture site. (Fig. 8B)

**Fig. 9:**
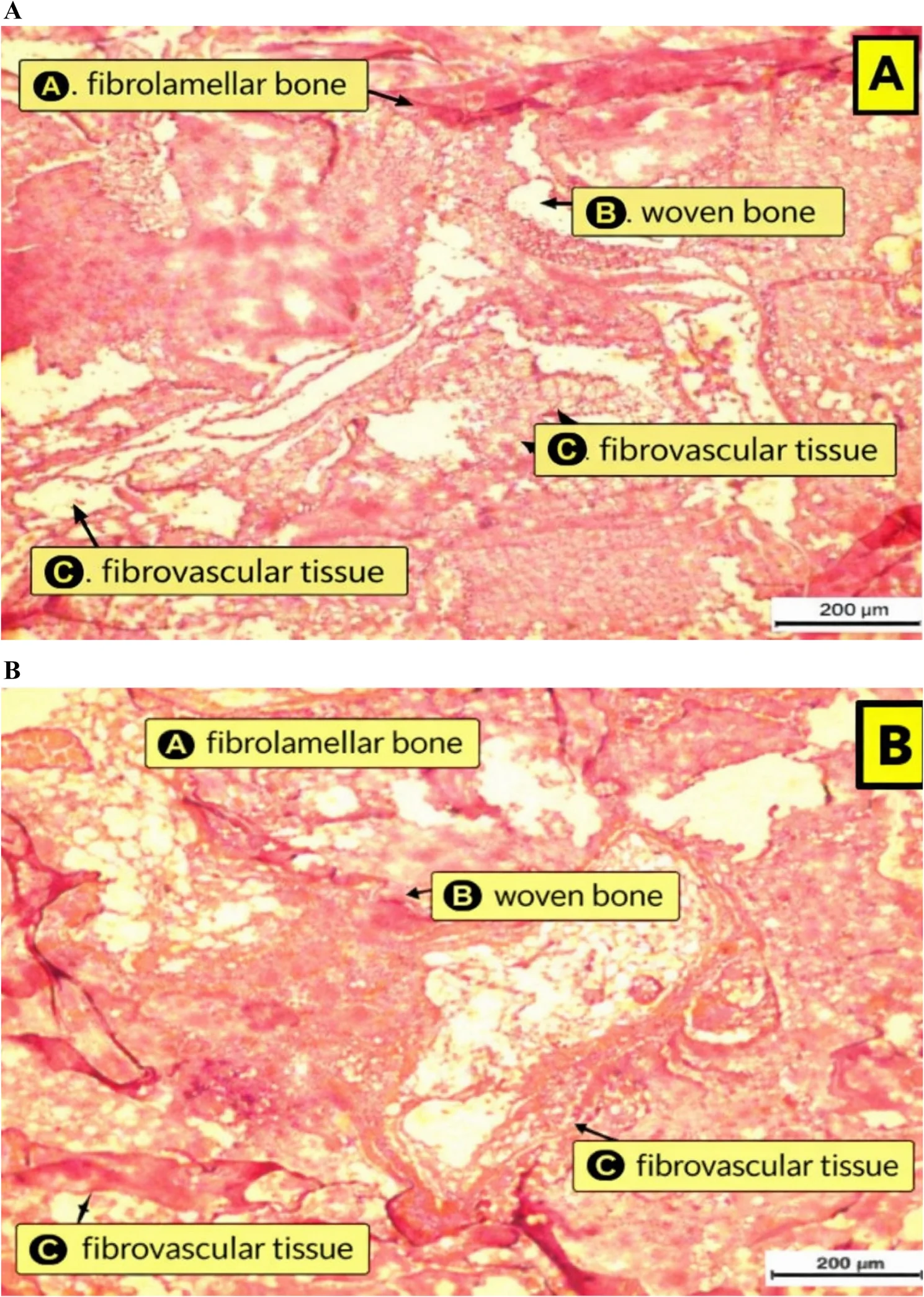
**A** Histological evaluation of bone healing at the fracture site at 12 weeks in group A. Histological sections stained with Hematoxylin and Eosin (H&E). Images were captured at ×100 magnification. Orientation: cranial aspect at the top, caudal aspect at the bottom. **B** Histological evaluation of bone healing at the fracture site at 12 weeks in group A and group B. Histological sections stained with Hematoxylin and Eosin (H&E). Images were captured at ×100 magnification. Orientation: cranial aspect at the top, caudal aspect at the bottom

At 12 weeks postoperative there Large sections of woven bone interspersed with a few islands of mature lamellar bone made up the whole fracture zone. Immature bone containing numerous irregularly distributed osteocytes was observed. The transition between lamellar bone and newly formed bone appeared indistinct (Fig. 9).

**Fig. 10:**
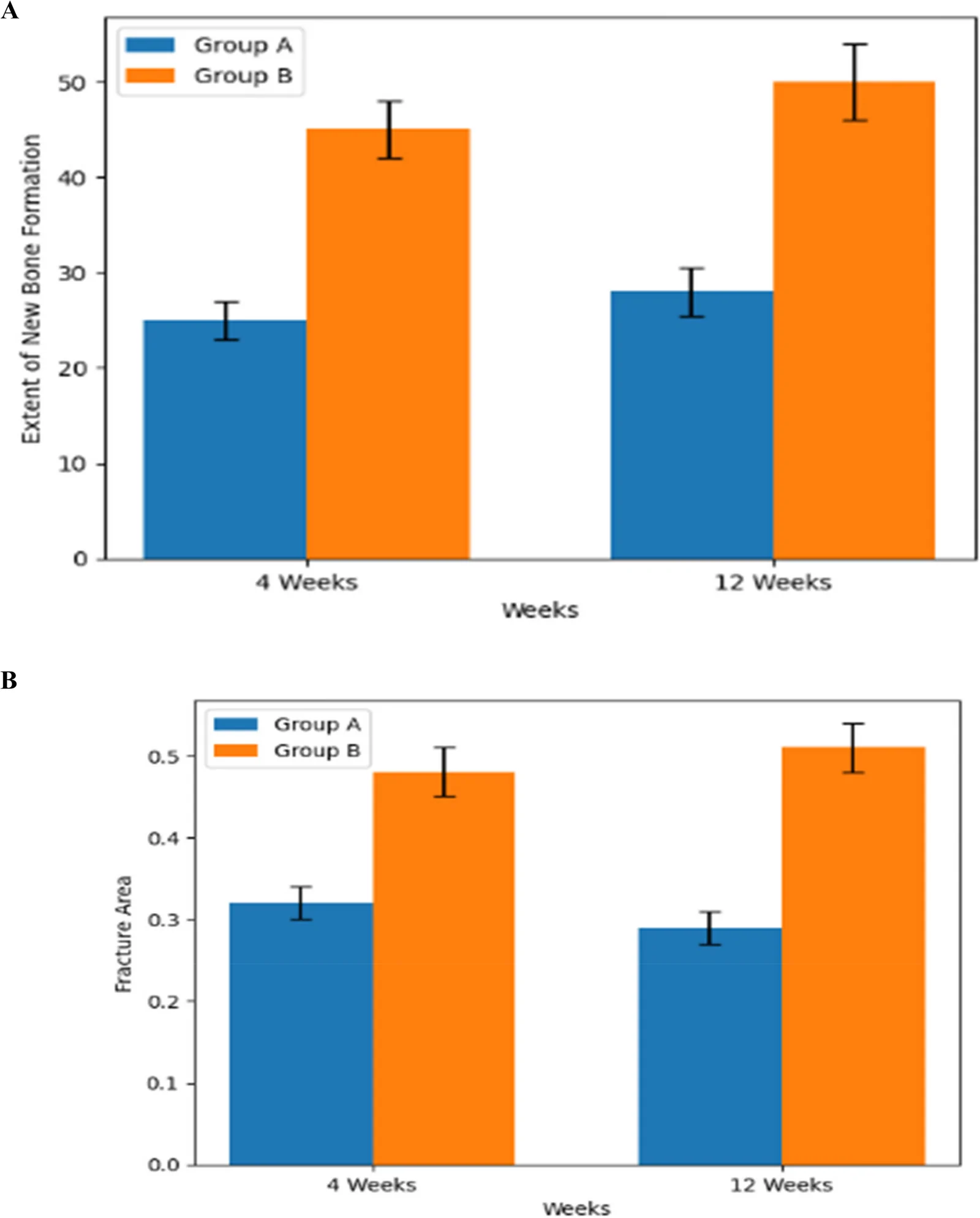
**A** comparison between the studied groups as regards new bone formation at 4 and 12 weeks. **B** comparison between the studied groups as regards area of fracture at 4 and 12 weeks

Table 1 shows that the extent of new bone formation was significantly lower in group A than in group B at week 4 (26.78 ± 4.3 vs. 48.4 ± 10.3, *P* < 0.005) and week 12 (28.119 ± 10.507 vs. 51.109 ± 11.348, *P* < 0.005). The surface area of the fracture was also significantly lower in group A than in group B (0.317 ± 0.065 vs. 0.485 ± 0.105 respectively in postoperative week 4 *P* < 0.005 and 0.287 ± 0.105 vs. 0.511 ± 0.115 respectively in postoperative week 12 *P* < 0.005) (Table 2) represents a Post hoc analysis of the sample that shows that the observed effect sizes (Cohen's d) for the extent of new bone formation at 4 weeks and 12 weeks was 2.74 and 2.10 sucessively, and the effect size for area of fracture at 4 weeks and 12 weeks was 1.92 and 2.03 sucessively, indicating large between-group effect sizes (Fig. 10).

**Table 1:** Comparison between the studied groups as regards histomorphometric assessment

		Group A No = 8	Group B No = 8	t	*p*
4 weeks	extent of new bone formation	26.78 ± 4.3	48.4 ± 10.3	5.4	< 0.0001 (HS)
	area of fracture (µm^2^)	0.317 ± 0.065	0.485 ± 0.105	3.4	0.0018 (HS)
12 weeks	extent of new bone formation	28.119 ± 10.507.	51.109 ± 11.348.	4.2	0.0009 (HS)
	area of fracture (µm^2^)	0.287 ± 0.105	0.511 ± 0.115	4.06	0.0012 (HS)

**Table 2:** Post hoc power analysis results

Variable (time point)	Mean difference	Cohen’s d	Statistical power (1 − β)
New bone formation (4 weeks)	**21.62**	**2.74**	**0.894**
Area of fracture (4 weeks)	**0.168**	**1.92**	**0.624**
New bone formation (12 weeks)	**22.99**	**2.10**	**0.699**
Area of fracture (12 weeks)	**0.224**	**2.03**	**0.671**

*P﻿* value for all evaluated variables at both time points was less than 0.05 which is considered significant

**Fig. 11:**
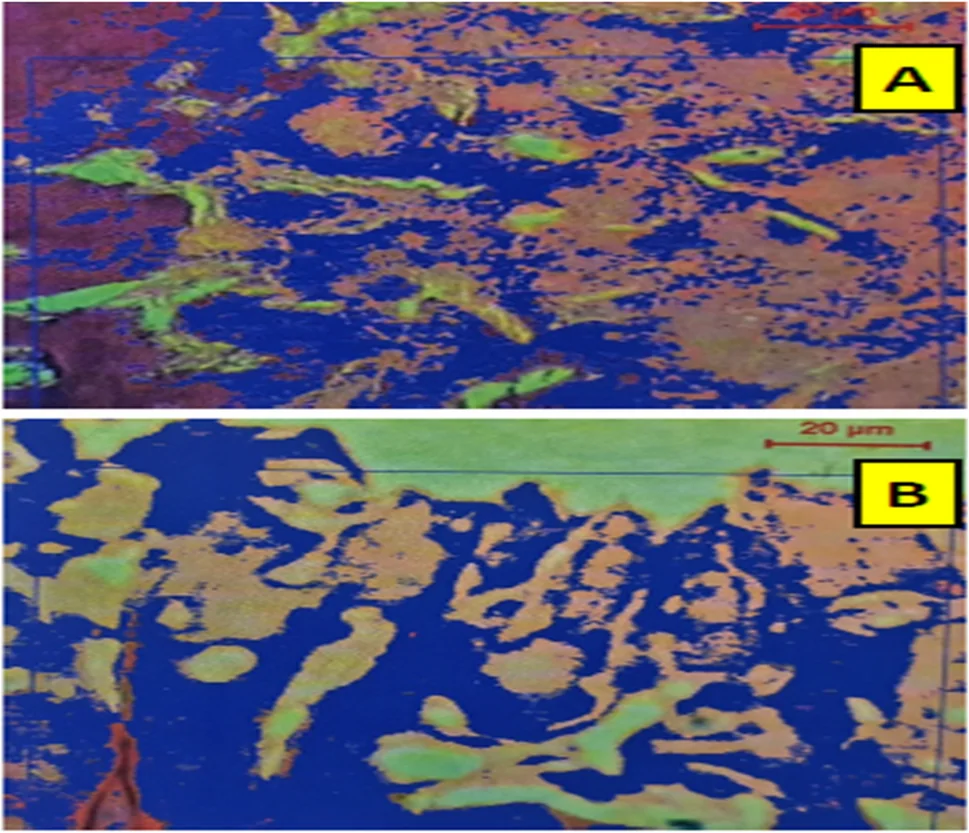
Analysis of group (A) 4 weeks postoperative. **a** and (**b**) new bone formation (Blue color) with pre-existing bone (Red color)

**Fig. 12:**
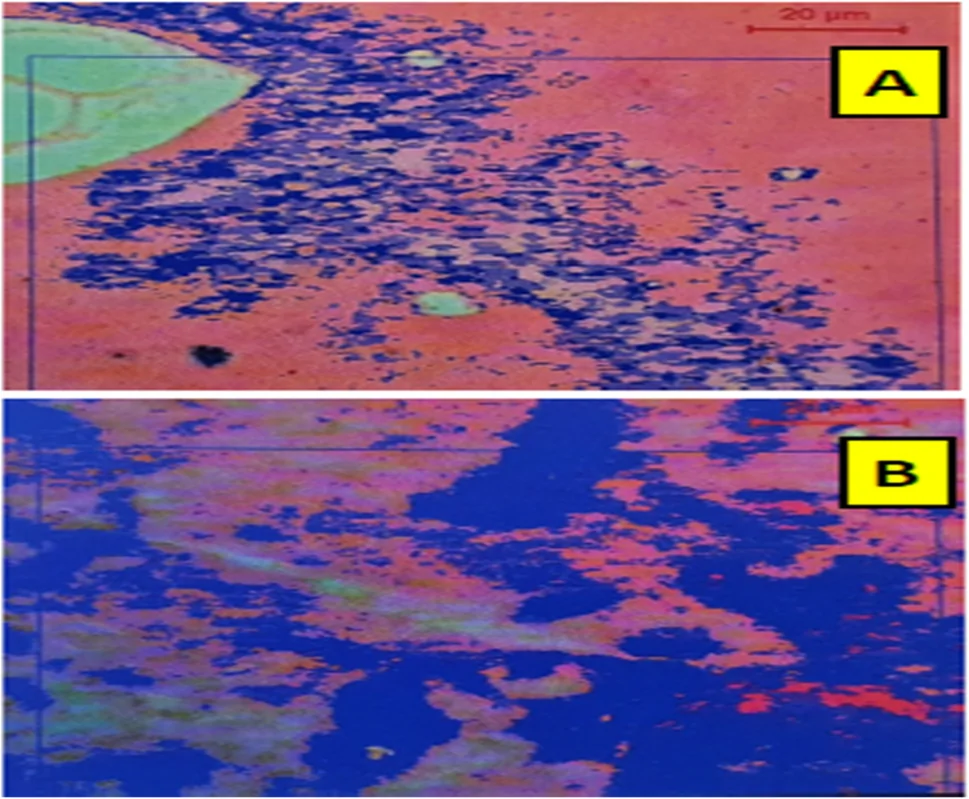
Analysis of group (A) 12 weeks postoperative. **a** and (**b**) medium new bone formation (Blue color) with pre-existing bone (Red color)

The histomorphometric analysis is shown in Figs. 11 and 12.

## Discussion

The experimental current study was conducted on sixteen mature male Mongrel dogs (ranged from 1 to 2 years old), weighting about 15–20 kg each with a linear fracture that was done under general anesthesia using surgical sagittal saw with aid of mallet and chisel in the right side of the mandible in the premolar molar area for each animal of the study, they were divided into two groups randomly. Group A was applied by 3D rectangular grid plate, while group B was applied by two locking plates. The dogs in this study were chosen as representative for humans because the canine mandible remains one of the most established and ethically acceptable large-animal models for evaluating maxillofacial implants because of its comparable bone density, size, and capacity to withstand functional loading. Thus, the model provides valuable translational information despite inherent anatomical differences. Moreover, using animals with similar characteristics and standardized housing, diet, and surgical protocols minimized physiological variability. While some inherent biological differences cannot be fully eliminated, the uniform handling reduces the impact on outcome interpretation.

Fixation Methods deeply impacts fracture stability and hence healing Rigid Fixation (Plates & Screws) provides strong biomechanical support, reducing micromovements at the fracture site and allowing primary bone healing (direct bone formation without callus formation). Miniplates & Lag Screws give enough biomechanical support yet allows some flexibility, which can be useful in some cases to induce secondary bone healing (callus formation) [10].

The fixation systems differed in more than one variable; plate design and screw type, however, the comparison reflects real-world clinical scenarios, where plate geometry and screw configuration are integral features of each system. The purpose of the study was to evaluate the overall performance of each commercially available system rather than isolate specific design parameters.

In this study a surgically created standardized fracture was done to avoid confounding variability and enable controlled comparison between fixation methods. Although real traumatic fractures can be more complex, controlled defects are widely accepted in experimental studies and ensure scientific reproducibility.

The surface area of the fracture has a significant effect on both healing and fixation method. A larger fracture surface gives more space for osteogenic activity, enhances healing, and improves blood supply delivering essential nutrients and oxygen to the healing site. Smaller fracture surfaces may slow healing due to limited osteogenic activity. There’s a higher risk of displacement, necessitating rigid fixation. Rigid Fixation (Plates & Screws) is essential for large fractures like mandibular angle fracture surfaces to prevent micromovements while miniplates & lag screws suits small fractures like parasymphseal fractures with moderate stability [11].

Bone histomorphometry is a quantitative histological method used to examine bone biopsies, providing detailed information about bone remodeling and structure. By using labeling agents administered before the procedure, which accumulate at sites of bone formation, this technique allows for dynamic analysis. It is a crucial research tool in the study of bone metabolism, offering insights that cannot be obtained through other methods [12].

A post hoc power analysis was performed to evaluate the adequacy of the sample size. The calculated statistical power for all evaluated variables exceeded 0.93, which is above the commonly accepted threshold of 0.80, indicating that the study had sufficient power to detect significant differences between the two groups.

The results of our study revealed that subjects in group A had significantly lower new bone formation in histomorphometric analysis along with significantly narrower surface area of the fracture. These findings indicate that fixation with 3D rectangular grid plates promotes primary intention bone healing, characterized by direct and organized bone formation with minimal callus and smaller fracture surfaces. In contrast, locking plates, while effective for fracture fixation, are associated with slightly greater interfragmentary movement, resulting in increased callus formation and a broader fracture surface. Thus, the differences observed likely reflect distinct biological healing responses rather than differences in mechanical performance. This comes in agreement with Mahmoud and Habaka, who concluded that the 3D miniplates provide better internal fixation treatment of mandibular para-symphyseal fractures [13].

Our findings are consistent with experimental animal studies in dog models of mandibular osteotomy by Elrewany et al., where 3D plate fixation resulted in increased new bone formation at early time points compared with conventional miniplates. These results support the concept that closer fracture segment approximation and favorable interfragmentary conditions can directly influence histologic indicators of primary bone healing [14].

Our results also align with emerging clinical data from prospective studies comparing various 3D plate configurations, which indicate that both standard and locking 3D miniplates support satisfactory bone healing and functional recovery in mandibular fractures, highlighting the contribution of 3D design to favorable clinical outcomes [15].

Similarly, retrospective clinical analyses of large cohorts treated with 3D miniplates have shown low overall complication rates and effective fracture reduction, suggesting that the 3D design supports favorable postoperative bone healing—consistent with our observed trend toward primary intention healing with reduced callus formation [16].

This also comes in agreement with Elsayed et al., who found that Miniplates and microplates gave functionally well-balanced fixation, satisfactory fracture reduction, restoration of occlusion, progressive radiographic bone healing, and the absence of hardware failure following fixation with 3D rectangular grid plates in mandibular angle fractures [17]. These findings are further supported by the large retrospective analysis by Bohner et al., who evaluated 336 patients with 391 mandibular fractures treated using 3D miniplates and reported low rates of major complications together with predictable fracture healing [18]. Moreover, a meta-analysis by Liu et al., which included 13 clinical studies involving 593 patients, concluded that 3D miniplates were associated with significantly lower rates of malocclusion and hardware failure than conventional miniplates, while infection, nonunion, and wound complications were comparable between the two fixation systems. Although these investigations primarily assessed clinical and radiographic outcomes rather than histological healing, their results complement the present histomorphometric findings by suggesting that the favorable biological healing response observed in the 3D rectangular grid plate group is associated with predictable and successful clinical outcomes [19].

The present findings are further supported by larger experimental animal studies that have investigated the biological response to different mandibular fixation systems using histological assessment. In a controlled goat model involving 24 animals, Kreshanti et al. demonstrated that an interlocking three-dimensional miniplate resulted in significantly enhanced early bone healing and improved biocompatibility compared with a standard miniplate system, as evidenced by greater fibrocartilage formation during the early healing phase and reduced inflammatory cell infiltration at later time points. Although both fixation systems ultimately achieved satisfactory bone remodeling, the interlocking three-dimensional plate promoted a more favorable early biological healing response [20]. Similarly, Freitag and Landau, in a longitudinal histological study of 40 canine mandibular fractures treated with either rigid compression plates or semirigid miniplates, demonstrated that fracture healing patterns are closely related to the stability provided by the fixation system, with more direct bone healing observed under conditions of greater mechanical stability and more callus-mediated repair occurring when limited interfragmentary movement was present. These experimental observations are consistent with the present histomorphometric findings, which suggest that the reduced new bone formation and narrower fracture surface observed in the 3D rectangular grid plate group are compatible with a more direct pattern of bone healing [21]. However, because the present study did not include biomechanical testing, these histological findings should be interpreted as reflecting differences in the biological healing response rather than definitive evidence of superior mechanical performance.

Not all experimental animal studies have reported identical patterns of bone healing following 3D rectangular plate fixation. Sverzut et al. observed delayed maturation of bone repair and persistence of fibrous connective tissue in certain regions of canine mandibular osteotomies treated with miniplates [22]. Similarly, Huh et al. demonstrated that single miniplate fixation or the use of monocortical screws alone was insufficient to provide predictable stability in canine mandibular continuity defects, whereas double miniplates with bicortical fixation produced more reliable healing [23]. These findings differ from the present results; however, direct comparison should be made cautiously because both studies employed substantially different experimental models. Sverzut et al. evaluated healing following a continuous mandibular osteotomy with regional histological assessment, whereas Huh et al. investigated critical-sized continuity defects rather than standardized fractures [22, 23]. In contrast, the present study used a standardized linear fracture model with controlled reduction and fixation, thereby minimizing variability related to fracture configuration. Consequently, differences in defect type, fixation protocol, loading conditions, and histological assessment methods may explain the apparent discrepancies between studies rather than indicating conflicting biological effects of the fixation systems.

## Conclusion

The present study found obvious differences in bone healing patterns between 3D rectangular grid plates and locking plates. Specimens treated with 3D grid plates showed a tendency toward reduced new bone formation and smaller fracture surface areas, while those treated with locking plates demonstrated relatively greater callus formation. However, the limitations of the study, including the small sample size, the use of a canine model, the relatively short follow-up period, and the lack of clinical and functional evaluation. Accordingly, no definitive conclusions regarding the underlying healing mechanisms can be drawn.

Within these constraints, the study provides preliminary comparative data on biological responses to different fixation methods. Further studies—particularly in human subjects, with larger sample sizes, longer follow-up, and comprehensive clinical assessment—are required to clarify and confirm these observations.

## Study limitations

The study provides a controlled experimental comparison between 3D rectangular grid plates and conventional locking plates, focusing on detailed histological and histomorphometric analysis of bone healing, which is rarely reported in previous mandibular fracture research. By using a homogeneous animal population and standardized surgical procedures, the study minimizes biological variability and allows a clear evaluation of the relative performance of each fixation system. These findings contribute to the existing literature by demonstrating the histological advantages and biomechanical rationale of 3D rectangular plates, providing preclinical evidence that may guide future clinical applications and studies in human mandibular fracture management. However, the study has the following limitations;

1. The sample size in each group was relatively small and no formal power analysis was performed; however, it is comparable to established large-animal fracture models and reflects ethical constraints associated with canine experimentation. The exclusive use of male mongrel dogs within a defined age and weight range, along with incomplete randomization, may limit generalizability, although employing a homogeneous animal population helped reduce biological variability and ensured consistent baseline bone quality. Additionally, the canine mandibular model does not fully replicate human mandibular biomechanics, highlighting the need for further validation in human clinical studies. While individual biological variability was not quantitatively assessed, standardized handling and random allocation were used to minimize its potential influence on the outcomes.
2. When the actual sample size of four animals per group at each time point was considered, the corresponding estimated statistical power ranged from 0.624 to 0.894. These findings indicate that the observed differences were associated with large effect sizes; however, the small number of animals per group limits the precision and statistical power of the analyses. Therefore, these findings should be interpreted cautiously and should not be considered evidence that the sample size was definitively sufficient.
3. We acknowledge that the follow-up period was relatively short for mandibular fracture studies. The selected duration was intended to evaluate initial bone healing, which were the primary objectives of this study. This limitation has been discussed, and longer follow-up periods are recommended in future investigations to assess long-term remodeling and functional outcomes.
4. The Study limited to one fracture location and the fracture was standardized. This was intended to minimize confounding factors, however we recommend further studies on traumatic fractures in different anatomical sites.
5. The clinical and radiographic evaluation are lacking to help us focus on histological evaluations we recommend further human studies were clinical and radiographic evaluation is mandatory.
6. The Differences in plate design and screw type and their effect on the study results. The purpose of the study was to evaluate the overall performance of each commercially available system rather than isolate specific design parameters.
7. The histological evaluation was not blinded, the examination was done by single histo-pathologist to minimize bias, however, we recommend further blind studies. Furthermore, the histological figures should be considered representative rather than definitive, as some images may not clearly demonstrate all annotated structures with optimal resolution. Although evaluations were performed using standard criteria by experienced examiners, this limitation may affect the visual interpretability of certain features. Therefore, the histological findings should be interpreted in conjunction with the overall qualitative assessment and supporting histomorphometric data.
8. No evaluation of implant failure, however, we recognize the value of detailed failure analysis and aim to integrate fatigue and stability testing in future long-term studies.
9. The surgical procedure was done by single surgical team. Further multicenter studies are recommended to investigate the effect of different surgical teams’ experiences and abilities on the outcomes of the procedure.

## Data Availability

The datasets used and/or analysed during the current study are available from the corresponding author on reasonable request.

## Abbreviations

ORIF: Open reduction and internal fixation
IMF: Inter-maxillary fixation
REC: Research ethics committee
